# Factors associated with physiotherapy provision in a population of elderly nursing home residents; a cross sectional study

**DOI:** 10.1186/1471-2318-7-7

**Published:** 2007-04-04

**Authors:** Chantal J Leemrijse, Marike E de Boer, Cornelia HM van den Ende, Miel W Ribbe, Joost Dekker

**Affiliations:** 1 NIVEL (Netherlands Institute for Health Services Research), Utrecht, The Netherlands; 2 Department of Nursing Home Medicine, Institute for Research in Extramural Medicine, VU University Medical Centre Amsterdam, The Netherlands; 3 Rheumatology centre St. Maartenskliniek, Nijmegen, The Netherlands; 4 Department of Nursing Home Medicine, Institute for Research in Extramural Medicine, VU University Medical Centre Amsterdam, The Netherlands; 5 Department of Rehabilitation Medicine, Institute for Research in Extramural Medicine, VU University Medical Centre Amsterdam, The Netherlands

## Abstract

**Background:**

Although physiotherapy (PT) plays an important role in improving activities of daily living (ADL functioning) and discharge rates, it is unclear how many nursing home residents receive treatment. Furthermore, there is a lack of insight into the determinants that influence the decision for treatment. In this study, we investigated how many nursing home residents receive PT. In addition, we analysed the factors that contribute to the variation in the provision of PT both between nursing homes and between residents.

**Methods:**

A random sample of 600 elderly residents was taken from a random sample of 15 nursing homes. Residents had to be admitted for rehabilitation or for long-term care. Data were collected through interviews with the nursing home physician and the physiotherapist. Multilevel analysis was used to define the variation in the provision of PT and the factors that are associated with the question whether a resident receives PT or not. Furthermore the amount of PT provided was analysed and the factors that are associated with this.

**Results:**

On average 69% of the residents received PT. The percentage of patients receiving treatment differed significantly across nursing homes, and especially the number of physiotherapists available, explained this difference between nursing homes. Residents admitted to a somatic ward for rehabilitation, and male residents in general, were most likely to receive PT. Residents who were treated by a physiotherapist received on average 55 minutes (sd 41) treatment a week. Residents admitted for rehabilitation received more PT a week, as were residents with a status after a total hip replacement.

**Conclusion:**

PT is most likely to be provided to residents on a somatic ward, recently admitted for rehabilitation to a nursing home, which has a relatively large number of physiotherapists. This suggests a potential under-use of PT for long-term residents with cognitive problems. It is recommended that physiotherapists reconsider which residents may benefit from treatment. This may require a shift in the focus of physiotherapists from 'recovery and discharge' to 'quality of life and well-being'.

## Background

Approximately 3% of people aged 65 years and older live in one of the Netherlands' 334 nursing homes [1]. Dutch nursing homes are healthcare institutions for chronically sick people requiring permanent complex nursing care. The mean age of the residents' population is 81.5 years. Most nursing homes have separate wards for, rehabilitation, day care, long-term physical care, and for patients with psycho-geriatric problems. In contrast with nursing homes in most other countries, the staff includes specially trained nursing home physicians, nursing assistants, psychologists and allied health care professionals. All Dutch citizens are insured under the Exceptional Medical Expenses Act (AWBZ), which covers all nursing home expenses, irrespective of the resident's income or personal finances [1,2]. The most common reasons for admission to a nursing home are long-term institutional care, rehabilitation, or special care, for example palliative care. One out of every three residents is discharged home after rehabilitation [1,2]., suggesting that rehabilitation services play an important role in the scope of community discharge. The intensive work delivered by the rehabilitation services in nursing homes is provided substantially by allied health-care personnel, mainly physiotherapists, but also occupational therapists and speech- and language therapists[3]. However, although allied health-care plays an important role in improving ADL functioning, discharge rates and survival rates, [4-12] it is unclear how many, or which residents, receive this kind of care in nursing homes. This study intends to fill this gap. It is known, however, that the percentage of nursing home residents receiving rehabilitation services differs substantially across nations and between nursing homes. Berg et al. found a prevalence of PT and/or OT ranging from 11% in the USA to 31% in Iceland [13]. In the UK, a prevalence of PT ranging from 6 to 10 % is reported [14,15]. Including only newly-admitted residents, Murray found the percentages of patients in the USA who received treatment (PT, occupational therapy (OT), speech and language therapy (SLT)) ranged from 50,5 % to 58%[7,16].

Furthermore, it is not only the number of residents receiving allied health care that is unknown. There is also a lack of insight into the determinants that influence the decision to treat, or explain the amount of care provided to patients in the nursing home. Several studies found that payment incentives were associated with the likelihood of receiving PT and OT, as well as with the total amount of treatment given [16-20]. Other studies indicate that residents with high cognitive functioning are more likely to receive treatment, suggesting that priority is given to residents with a higher 'functional status' [7,13,18,21]. Berg et al (1997) found that residents over the age of 85 were less likely to receive PT or OT, as were residents who had been in the nursing home for more than 90 days.

Until now, there is little information about how many, or which, residents receive PT in Dutch nursing homes. We expect that prevalence rates for rehabilitation services will vary across different nursing homes, as there is a lack of nationally accepted standards or criteria, on which to base decisions regarding the provision of treatment. Although health-care provision should be based on residents' needs, we hypothesise that the provision of treatment is associated with the characteristics of the residents, for example need for care or age, and too with characteristics of the nursing home, for example the type of nursing home and the supply of care.

Therefore, the aim of the study is twofold. Firstly, to study the variation in the provision of PT among nursing homes and the factors that are associated with this variation. Secondly, to study the variation in the amount of PT both among nursing homes and among residents, and to identify factors that are associated with this variation.

## Methods

### Sample

A cross-sectional design was used. Randomisation took place on two levels. Firstly, a random and weighted sample of 15 nursing homes was obtained from a list of all Dutch nursing homes [22]. These 15 nursing homes represent 5% of all Dutch nursing homes providing care exclusively for somatic residents (somatic nursing homes; N = 44) and nursing homes with both somatic and psycho-geriatric wards (combined nursing homes; N = 242). Stratification was made by the type of nursing home (somatic versus combined) and by the size of the nursing home. For this latter, somatic nursing homes were divided into homes with 100 beds or less (N = 21) and homes with more than 100 beds (N = 23). Combined nursing homes, which are generally larger, were divided into homes with 200 beds or less (N = 142) and homes with more than 200 beds (N = 100). Two somatic nursing homes were randomly selected, one with less and one with more than 100 beds. Thirteen combined nursing homes were randomly selected, 6 with less, and 7 with more than 200 beds (table 1).

Secondly, from these 15 nursing homes a random and weighted sample of 600 residents was taken, representing 1 % of all 51.174 beds available in Dutch (somatic and combined) nursing homes [22]. The residents were randomly selected by the researcher from a (anonymous) list of all residents. The number of selected residents per nursing home was weighted by the total number of beds destined for rehabilitation and long-term care in the involved nursing home. Only residents over 55 years of age were included, because the study was designed to focus on health-care for the elderly. Furthermore, residents had to be admitted to the nursing home for either rehabilitation services or for long-term institutional care. Patients consent was not needed according to the Dutch 'Regulations on medical research involving human subjects', since data were collected anonymously (using residents' numbers), and residents were not subjected to any treatment. As a consequence, there was no decline to participate.

### Data collection

Data from these residents were collected through interviews with the nursing home physician and the physiotherapist assigned to the resident. As part of these highly structured interviews, a questionnaire was filled out by the researcher for each resident, in the presence of the physician and therapist, enabling them to consult the medical file of the resident simultaneously. The domains in the questionnaire were based on the Minimum Data Set of the Residents Assessment Instrument (RAI)[23] and determined in mutual agreement with a number of nursing home physicians and physiotherapists. The following areas of the RAI were addressed: communication and hearing patterns, physical functioning and structural problems, mood and behaviour patterns, disease diagnoses, oral and nutritional status, skin conditions, cognitive patterns, vision patterns, and continence. All information was based on the residents' medical file and on the judgement of the nursing home physician and the physiotherapist. No additional measurements were taken.

### Outcome variables

Two outcome, or dependent, variables were used. The first, dichotomous, outcome variable was whether the resident had received PT in the six months preceding the interview. All therapeutic intervention as registered in the medical file, was designated as treatment given, without time restrictions on the amount of treatment per week. The second, continuous, outcome variable was the amount of PT provided to the resident during the last 6 months, estimated by the PT in minutes per week.

### Independent variables

The 'predictor', or independent variables were divided into two groups on the basis of their level, that is the characteristics on the level of the residents or characteristics on the level of the nursing homes (table 2). The medical diagnosis and co-morbidity, as determined by the nursing home physician, were taken into account on the level of residents' characteristics. The four medical diagnoses most frequently found among the 600 residents, stroke, dementia, status after total hip replacement, and non specified neurological disorders, were included in the analysis. Other diagnoses were treated as a reference group. When residents had more than one medical diagnosis, the diagnosis most determining the state of health, according to the nursing home physician, was used. Co-morbidity was defined as the number of medical diagnoses, excluding the main diagnosis. While 'co-morbidity in functioning' was defined as the number of (additional) impairments and limitations in activities.

Furthermore, we asked the nursing home physician which impairments or limitations had the most impact on the residents' health status. The impairments mentioned were categorised as 'impairments in mental functions' or as 'limitations in mobility and self-care'. Impairments and limitations in activity that could not be classified into one of these two categories, such as impairments in functions of the skin, genitourinary functions and limitations in communication, were treated as a reference group.

Additionally, limitations in communication, limitations in behaviour, age, sex, the length of stay since admission, the reason for admission (rehabilitation versus long term care) and whether the resident was staying on a somatic or psycho-geriatric ward, were used as independent variables [24,25]. On the level of the nursing home characteristics the volume of available PT, measured as full time equivalents (FTE) per bed with 1 FTE being 36 hours a week, the type of nursing home, the presence of a stroke unit and the degree of urbanisation were taken into account [26,27].

### Data-analysis

Descriptive statistics for all variables were calculated with SPSS 11.5. Multilevel logistic regression analysis, carried out by MLwiN 1.1, was used to define the amount of variation in the provision of PT between nursing homes, and the factors that determine whether a resident receives PT or not. By means of multilevel linear regression, the amount of variation in the mean volume of PT between nursing homes and the factors that act on this volume were analysed. This latter analysis was carried out for the subgroup of residents that actually received PT. Multilevel analysis was used because the data had an intrinsic hierarchical nature. The sample of residents, level 1, is nested in the sample of nursing homes, level 2. The data, therefore, were not from independent observations, violating a major assumption of traditional regression analysis. In multilevel analysis, multiple levels are taken into account and in linear multilevel regression the proportion of variance at each level can be calculated. The order of adding predictor variables was determined by their level. The analysis was carried out in four steps:

1. Firstly, an intercept model was made, which is a model without any independent variables. This model estimates, in case of logistic regression, the mean percentage of the nursing home residents that receive allied health care. In case of a linear regression, the intercept model estimates the mean volume of therapy that is provided to the recipients. This latter model also establishes the contribution of both levels to the variation in the volume of treatment.

2. In the next step, all independent variables at the level of the residents were added. The contribution of each factor is expressed by the regression coefficient (β) and the standard error (SE).

3. In the third step, all independent variables on the level of the nursing home were added.

4. In the last step, only the factors that significantly contribute to the utilisation of PT are left in the model, that is the factors for which the quotient of β and SE is larger than |1.96|, the level of significance being 0.05.

For categorical independent variables indicator coding was used with the last category in each group treated as the reference group. The continuous independent variable residents' age was centred around the median age.

## Results

The mean age of the 600 residents included was 81,53 years (sd: 8.24). There were 391 women and 209 men. Most residents were admitted for long term institutional care (82.3%), and less than one fifth for rehabilitation (Table 3). More than half of the residents (n = 320) stayed on a somatic ward, and 278 on a psycho-geriatric ward. The mean length of stay in the nursing home since admission was 3.13 years (sd: 3.56).

Four hundred and four residents received PT (67.3%), on average 55 minutes a week. The medical diagnoses most frequently found were dementia (43.3%) and stroke (24.2%). Forty-four percent of the residents (44.3%) suffered most from limitations in mobility and self-care, while for 35.7% of the residents impairments in mental functions had the largest impact on their health status (Table 4).

Furthermore, 95% of the residents suffered from co-morbidity, the mean number of additional medical diagnoses being 3.52 (sd: 2.26). The mean number of additional impairments and limitations in activities was 3.45 (sd: 1.75), while 75% of the residents suffered from more than one impairment or limitation. Almost half of the residents had limitations in behaviour (49%) or communication (45%). Of the 15 nursing homes, nine were located in an urban region and six in a more rural environment. Two nursing homes only provided care to residents with somatic problems, while 13 homes also had psycho-geriatric wards. Only two nursing homes did not have a stroke unit.

### Provision of physiotherapy

#### How nursing homes differ in the percentage of residents receiving physiotherapy

On average 69% of the residents of the participating 15 nursing homes received PT (min-max: 39%-93%; interquartile range: 34.2). The percentage of residents receiving PT differed significantly across the 15 nursing homes, and this variance between nursing homes did not decrease after adding the independent variables on the residents' level. Adding the characteristics of the nursing homes itself, however, reduced the variance between nursing homes by 85%, the remaining difference being no longer significant. Apparently, the characteristics of the 15 nursing homes themselves contribute the most to the significant difference in the percentage of PT recipients among nursing homes.

#### Variables associated with whether a resident receives physical therapy or not

The factor most associated with the question whether a resident received PT in a nursing home was which ward they were on. Residents admitted for rehabilitation and those residents staying on a somatic ward received PT more often. Men were more often referred to PT than women. Furthermore, the amount of available PT personnel was associated with the chance of receiving treatment. The more FTE present in the nursing home, the more likely a resident was to receive PT. Co-morbidity enlarged the chance of receiving PT (Table 5). Finally, the chance of receiving PT slightly diminished as more time elapsed after admission.

### The volume of physiotherapy

#### How nursing homes differ in the volume of treatment offered per week

The residents referred to PT (N = 404), received 55 minutes of care a week on average (sd: 41). The amount of PT provided to a resident was not significantly different across nursing homes, although differences were considerable (min-max: 34–88 minutes; interquartile range: 15). Most variance in the volume of treatment was located among residents (93%), while 7% of the variance was found among nursing homes.

#### Variables that are associated with the amount of physical therapy

Just as with the provision of PT, the resident's ward is the factor most associated with the amount of treatment given. Residents admitted for rehabilitation received more PT per week compared to residents admitted for long-term institutional care. Residents recovering after a total hip replacement received more PT per week compared to residents with other diagnoses (Table 6). Furthermore, residents staying in a nursing home with somatic residents only, received less PT than residents of nursing homes that also provided psycho-geriatric care. Finally, residents who suffered mainly from limitations in mobility and self-care received more PT in contrast with residents suffering from other impairments and limitations, while the number of impairments and limitations, that is co-morbidity in functioning, is related negatively to the amount of PT.

## Discussion

### Differences among nursing homes

This study is the first one to examine how many Dutch nursing home residents receive PT and the extent to which the provision of PT varies across nursing homes. For this purpose, a random sample of nursing homes was taken, stratified for type and size of the nursing home, leading to representative representation of the population of Dutch nursing homes (table 1). The number of randomly selected residents was weighted by the total number of beds destined for rehabilitation and long-term care in the involved nursing homes. In addition, the proportion of residents living at a somatic ward and residents living at a psycho-geriatric ward in our sample, reflected the proportion of beds destined for somatic and psych-geriatric care in the Netherlands (table 1).

On average 69% of the nursing home residents received PT. In comparison with the international literature this is a rather high percentage of the nursing home population. This may, however, be partly due to different definitions of receiving treatment. In this study, all physiotherapeutic care as registered in the medical file, was designated as PT, without time restrictions for the amount of treatment per week. Adopting a minimum restriction of 30 minutes PT a week, as was used by Berg et al (1997) in some of the investigated countries, would result in 50% residents being treated [13]. Furthermore, this high percentage of treatment recipients is probably due to the fact that physiotherapists are almost always employed by Dutch nursing homes, making referral easier. However, the percentage of residents receiving PT ranged significantly from 35% to 90%, which reflects a disparity in the care far beyond our expectations. Since we controlled for residents' characteristics, this difference can not be explained by a variation in the characteristics of the nursing home population as far as is measured in this study. In contrast, the availability of PT, varying from 1.12 to 3.33 FTE per 100 beds, contributes significantly to the difference in the percentage residents receiving treatment among nursing homes. Nursing homes, together with the nursing and rehabilitation facilities they provide, are mainly covered by the Exceptional Medical Expenses Act (AWBZ). In addition, residents contribute to the nursing home, depending on their personal financial resources or income. Inequalities in the financial resources of nursing homes may therefore explain some of the differences in the availability of physiotherapists, but we expect that divergent management policies of the nursing homes also play a role. Allied health care facilities will not always be given priority by management.

### Differences among residents

In this study the medical diagnosis was not associated with whether or not a resident receives PT. This finding is, however, somewhat biased by the fact that most residents with dementia stay on a psycho-geriatric ward, leading to a high correlation between the factor 'dementia' and the ward a residents is staying on. So when the factor 'somatic ward' is left out of the analysis, residents with dementia are less likely to receive PT, leaving all other predictor variables unchanged. Furthermore, residents admitted for rehabilitation and residents living on a somatic ward were more likely to receive PT than residents admitted for long term care or residents living at a psycho-geriatric ward, irrespective of their medical diagnoses. While it may be obvious that residents admitted for rehabilitation need physiotherapeutic care, it carries the risk that residents admitted for long-term institutional care do not receive the treatment they might need, because therapists assume that treatment is less effective for this group and residents admitted for rehabilitation have precedence in cases of limited availability of PT. However, the studies by Arlings and by Przybylski (1996) revealed that increasing the amount of PT and OT did have a positive effect on the functional status of long-term care residents [8]. The pattern found in our study, that residents living at a psycho-geriatric ward receive PT less often (table 6), also reflects the belief that especially residents with good cognitive functioning are likely to benefit from treatment, whereas several studies demonstrate that geriatric patients with cognitive dysfunction show similar gains in functional status following specialised rehabilitation [4,28,29].

So despite the fact that there is evidence that PT can be effective for long-term residents and for residents with low cognitive functioning, these residents receive less treatment. This suggests an under-use of PT for long-term residents and residents with low cognitive functioning. It is, therefore, recommended that physiotherapists working in nursing homes carefully reconsider which residents may benefit from treatment. This may need a shift in the focus of the physiotherapist, and other rehabilitation disciplines, from 'recovery and discharge' to 'quality of life and well-being' of residents. The relevance of such a change of philosophy is illustrated by the fact that a relatively large proportion of elderly residents who have been admitted for rehabilitation, will not be discharged home again and eventually remains at the nursing home receiving long-term care.

In our study, men were more likely to receive PT than women, irrespective of their medical condition. The reasons for this can only be hypothesised. Since most women survive their husbands, men may be more motivated for treatment and have more opportunities to return home, if their wife is capable of providing the necessary care and social support [30]. However, the actual reasons remain unclear and the little literature available on gender differences regarding PT in nursing homes reveal conflicting findings. Whereas Berg et al (1997) found that men were less likely to receive rehabilitation services in Italian nursing homes, Yeh (2004) and Kupper (2006) found significantly more men among physiotherapy recipients of PT than could be expected by chance alone. Obviously, further research is needed to explain these gender differences.

This study has some limitations. It was not possible to take the severity of the residents' medical problems and impairments into account, due to the method of gathering data. Data were collected through interviews with the nursing home physician and the physiotherapist, and were based on the residents' medical file. The severity of the patient's condition or their functional status were not asked, since these aspects are not always registered in a standardised manner, making a comparison between different nursing homes difficult. There are, however, no reasons to expect systematic differences in the severity of the medical problems between the residents populations of the 15 nursing homes. Another limitation is that the accuracy of assessing the medical diagnosis and the presence of impairments in mental functions and limitations in mobility and self-care, relies on the nursing home physician reports. Since diagnostic procedures used for establishing the residents' health problems were not standardised, they may differ between nursing homes.

## Conclusion

The chance of nursing home residents receiving PT differs significantly among nursing homes. This difference between nursing homes is highly associated with the difference in supply of physiotherapists. Also within the nursing home the opportunities for receiving PT are unequal, while it is most likely provided to male residents with good cognitive functioning who have recently been admitted for rehabilitation. Without wanting to deny the great need of most residents admitted for rehabilitation for physiotherapeutic treatment, this suggests a potential under-use of PT for specifically female long-term residents with cognitive problems. Since there is evidence from the literature that residents with cognitive problems and residents receiving long-term care may also benefit from PT, these groups deserve attention from PT services. Treatment goals for these residents may be formulated better in terms of quality of life and well-being instead of recovery and discharge. Furthermore, more insight is needed into the residents' needs and the efficiency of the care provided, in order to establish the optimum amount of PT for nursing home residents. This applies to both candidates for rehabilitation and for residents admitted for long-term care.

## Competing interests

The author(s) declare that they have no competing interests.

## Authors' contributions

CL, MEdB, CHMvdE, MWR and JD contributed to the design of this study and the main ideas of this paper. MEdB organised and performed the data-collection. CL performed the statistical analysis and drafted the manuscript. All authors read and approved the final manuscript.

## Pre-publication history

The pre-publication history for this paper can be accessed here:



## Figures and Tables

**Table 1:** Number of nursing homes and beds destined for somatic and psycho-geriatric care of the study sample, versus the number of nursing homes and beds in the Netherlands

		**Dutch population N(%)**	**Study sample N(%)**
**Somatic nursing homes**^**1**^	≤ 100 beds	22 (50.0)	1 (50.0)
	> 100 beds	22 (50.0)	1 (50.0)
	Subtotal	44 (14.8)	2 (13.3)
**Combined nursing homes**^**2**^	≤ 200 beds	142 (58.7)	7 (53.8)
	> 200 beds	100 (41.3)	6 (46.2)
	Subtotal	242 (85.2)	13 (86.7)
	Total	284 (100.0)	15 (100.0)
**Beds for somatic care**		26356 (51.5)	320 (53.3)
**Beds for psycho-geriatric care**		24818 (48.5)	278 (46.3)
	Total	51174 (100.0)	598 (99.7)*

^1 ^= Nursing homes providing exclusively somatic care

^2 ^= Nursing homes with both somatic and psycho-geriatric wards

* = of 2 residents information on the ward is missing

**Table 2:** Outcome variables and independent variables used in the multilevel analysis to study the variation in the provision of PT among nursing homes and residents, and the factors that are associated with this variation

**Variables**	**Measurement**	**in analysis as:**
**Outcome variables**		
Provision of PT	Yes, No	Category
Amount of PT	Minutes/week	Continuous
**Characteristics at the level of the residents**		
Stroke	Yes, No	Category
Dementia	Yes, No	Category
Total hip	Yes, No	Category
Non specified neurological disorders	Yes, No	Category
Other (reference)	Yes, No	Category
Limitations in mobility and self-care	Yes, No	Category
Impairments in mental functions	Yes, No	Category
Other impairments (reference)	Yes, No	Category
Co-morbidity medical diagnoses	Number of diagnoses – 1	Continuous
Co-morbidity in functioning	Number of impairments -1	Continuous
Impairments in communication (incl. aphasia)	Yes, No	Category
Limitations in mood/behaviour	Yes, No	Category
Sex	Man, Woman	Category
Age	Date of birth	Continuous in years, corrected for median age
Ward	Somatic, PG	Category
Time elapsed since admission	Date of admission	Continuous in years
Reason for admission	Rehabilitation, long term care	Category
**Characteristics at the level of the nursing home**		
Type of nursing home	Somatic nursing home, combined nursing home (somatic and PG-wards)	Category
Urbanisation	ZIP code; urban versus rural	Category
Stroke unit	Yes, No	Category
FTE	FTE/100 beds	Continuous, rank

PT = Physical therapy

FTE = full time equivalent

PG = psycho-geriatric

**Table 3:** Reason for admission and residence ward of the sample of residents (n = 600)^1^

	**Somatic ward**		**Psycho-geriatric ward**		**Total**	
			
	N	%	N	%	N	%
**Rehabilitation**	103	32.2	3	1.1	106	17.7
**Long-term care**	217	67.8	275	98.8	492	82.3
						
**Total**	320	100.0	278	100.0	598	100.0

^1 ^Missing data ward and reason for admission: n = 2

**Table 4:** Residents' medical diagnoses and impairments most determining the residents health status, according to the nursing home physician (n = 600)

**Medical diagnosis most determining the residents health status^1^**	**N**	**%**
Dementia	235	43.4
Stroke	131	24.2
Other neurological disorders (including Parkinson's disease)	38	6.7
Total hip	20	3.7
Other	162	22.0

**Impairments most determining the residents health status**	N	%

Impairments in mental functions	266	44.3
Limitations in mobility and self-care	214	35.7
Other	120	20.0

^1^14 medical diagnoses are missing

**Table 5:** Factors significantly associated with the provision of physiotherapy, found with logistic multilevel analysis (N = 600)

	**β**	**SE**	**Odds Ratio**	**95% CI**	
Intercept	-1.55	0.34			
**Factors at residents' level**					
Male	0.50	0.22	1.65	1.07;	2.53
Co-morbidity	0.11	0.05	1.11	1.01;	1.22
Length of stay (years)	-0.06	0.03	0.94	0.89;	1.00
Rehabilitation (ref. Long-stay)	3.49	1.02	32.69	4.41;	242.29
Somatic ward (ref. PG ward)	1.12	0.22	3.07	2.00;	4.71
**Factors at nursing home level**					
FTE physiotherapist	0.17	0.03	1.19	1.12;	1.26

**Table 6:** Factors significantly associated with the amount of physiotherapy in minutes per week found with linear multilevel analysis (analyzed only for residents receiving PT; N = 404).

	**β (SE)**
Intercept	49.85 (5.16)
**Factors at residents' level**	
Rehabilitation	44.98 (4.23)
Total Hip	22.35 (8.48)
Mainly limitations in mobility and self-care	9.16 (3.64)
Co-morbidity in functioning	-2.70 (1.00)
**Factors at nursing home level**	
Somatic nursing home	-21.33 (8.50)
